# Performance‐enhancing substances in sport: A scientometric review of 75 years of research

**DOI:** 10.1002/dta.3677

**Published:** 2024-03-16

**Authors:** Alessandro Carollo, Ornella Corazza, Maria Mantovani, Nicolò Silvestrini, Olivier Rabin, Gianluca Esposito

**Affiliations:** ^1^ Department of Psychology and Cognitive Science University of Trento Rovereto Italy; ^2^ School of Life and Medical Sciences University of Hertfordshire Hatfield UK; ^3^ World Anti‐Doping Agency Montreal Québec Canada

**Keywords:** CiteSpace, doping, fair sport, performance‐enhancing substances, scientometric review

## Abstract

The use of performance‐enhancing substances not only undermines the core values of sports but also poses significant health risks to athletes. In a fast‐evolving doping environment, where sport professionals are constantly seeking novel and illegal means to bypass doping tests, and new substances are regularly detected on the drug market, it is crucial to inform authorities with updated evidence emerging from scientific research. The current study aims to *(i)* outline the structure of knowledge in the literature on performance enhancers in sports (i.e., most active countries, main sources, most productive authors, and most frequently used keywords); *(ii)* identify the most impactful documents in the field; and *(iii)* uncover the main domains of research in the literature. To do so, we conducted a comprehensive scientometric analysis of the literature on doping, sourcing our data from Scopus. Our research involved a document co‐citation analysis of 193,076 references, leading to the identification of the 51 most influential documents and seven key thematic areas within the doping literature. Our results indicate that the scientific community has extensively studied the most prevalent doping classes, such as anabolic agents and peptide hormones, and little is still known about the use of contaminated supplements or other types of enhancers identified as emergent trends. Concurrently, technological advancements contributed to the development of more sophisticated doping detection techniques, using blood or urine samples. More recently, the focus has shifted towards the athlete biological passport, with research efforts aimed at identifying biomarkers indicative of doping. The dynamic nature of doping methods underlines the necessity for more robust educational campaigns, aiming at raising awareness among sports professionals and their entourage about the dangers of doping and the intricacies of its control mechanisms.

## Graphical abstract

The use of performance‐enhancing substances in sports undermines values and risks athletes' health. This study analyzes doping literature, identifying influential documents and research domains. While prevalent doping classes are well‐studied, emerging trends like contaminated supplements receive less attention. Technological advancements drive sophisticated detection techniques. The study emphasizes the need for awareness campaigns among athletes.

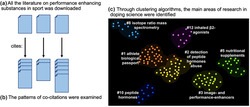

## 1 INTRODUCTION

Fair competition is the core principle of any sport activity. Athletes strive to win games and set records through their abilities and intense training. However, in some cases, they might opt for unfair shortcuts that do not align with the founding values and principles of sport. One of these is represented by doping (or performance‐enhancing drugs).^1^

The use of substances to alter sportive performances is not a new phenomenon. In ancient Greece and Rome, athletes used to combine diet modifications and natural potions to improve their physical fitness and performance in sports competitions.^2^ The use of stimulants to improve physical performance has not been limited to sportive activities. More recently, Zulu warriors assumed a spirit called “dope” as a stimulant before fights and even religious rituals.^3^ It seems that the name of the Zulu's beverage could have inspired the term “doping,” which appeared on an English dictionary in 1889 for the first time, and it referred to a narcotic potion used to reduce the performance of racehorses.^4^, ^5^ With the years, the meaning of the term “doping” was extended to cover all substances with stimulating properties.

Doping not only violates the founding principles of sport, but it often poses serious health hazards for athletes. As stated by Lippi et al.,^4^ doping has to be considered a multifaceted problem because it targets all bodily functions. In fact, abuse of performance enhancers reportedly increases the risk of cardiovascular^6^ as well as hematologic, psychiatric, neuropsychologic, hormonal, and metabolic side effects.^7^, ^8^ Moreover, the use of performance enhancers can lead to impairments in cognitive (e.g., visuospatial abilities) and emotional (e.g., anxiety, emotion regulation, and impulse control) functions.^9^, ^10^

With the aim of, first and foremost, safeguarding the health of athletes and, second, preserving the fairness of sport, in 1999, the World Anti‐Doping Agency (WADA) was established with an initiative led by the International Olympic Committee and some governments. WADA is an international independent agency in charge of developing, harmonizing, and coordinating anti‐doping rules and policies across all sports and countries.^11^ On a yearly basis, WADA publishes a list of prohibited substances and methods to inform athletes, coaches, and other professional figures in the sport environment about what is legal and when. The Prohibited List includes all substances and methods that meet at least two of the following three criteria: *(i)* it has the potential to enhance or enhances sport performance; *(ii)* its use represents an actual or potential health risk to the athlete; and *(iii)* it violates the spirit of the sport.^11^

In today's society, the use of performance enhancers is spread across all domains of life. Cultural pressures, fast‐paced lifestyles, and technological advancements have contributed to a growing willingness among individuals to use these substances to boost their professional and personal lives.^12^ This trend is further encouraged by marketing strategies that advertise several sport foods, vitamins, and supplements as “natural” and “safer” options in comparison with pharmaceutical products to improve not only performance but also people's physical appearance and well‐being.^13^ Research studies have revealed the extent of this trend. For instance, Corazza et al.^14^ showed that in fitness settings across Europe (i.e., the United Kingdom, Italy, the Netherlands, and Hungary), more than a third of the sample used fitness‐enhancing supplements without medical consultation. Additionally, the global survey by Dores et al.^15^ documented that the use of substances to enhance physical performance and appearance is also widespread across the general population, particularly among individuals who suffer from appearance anxiety, exercise addiction, and low self‐esteem. Such a phenomenon is concerning because, as in most of the cases, the use of enhancers remains under‐regulated and users might face a wide range of unwanted health risks, especially when the products they buy are contaminated with novel psychoactive substances (NPS) or other illicit drugs.^16^ The class of NPS includes all substances specifically engineered to mimic the effects of controlled substances and to bypass current drug control legislation.^17^ The use of NPS in sports is still an under‐recognized and under‐investigated issue in the scientific literature.^18^ It is worth noting that regulating NPS by anti‐doping authorities often leads to the synthesis and production of new NPS, making their regulation even more challenging.^19^

Because of the importance of the continuous dialog between the scientific community and regulatory organizations like WADA, the current study aims to provide an overview of the literature on performance enhancers in sports. In particular, the current study aims to *(i)* analyze the structure of knowledge (most active countries, journals, authors, and most frequently used keywords); *(ii)* identify the most impactful documents; and *(iii)* pinpoint the main domains of research in the literature on performance enhancers in sports. To do so, a scientometric approach will be adopted. Scientometrics is a field located at the intersection between bibliometric analysis (i.e., application of quantitative techniques to bibliometric data) and scientific mapping (i.e., visualization of the temporal evolution of a research domain).^20^, ^21^, ^22^ The current manuscript follows the approach presented in our recent publication, in which we used a scientometric approach to identify impactful documents and research domains in the literature on NPS.^17^

## 2 METHODS

### 2.1 Literature search

As a common practice in scientometric reviews,^17^ the literature search was performed on Scopus. Scopus was chosen because it offers wider coverage of indexed journals and documents as compared with Web of Science.^23^

All documents used for the analysis were retrieved in date March 9, 2023, by using the following search string: “TITLE‐ABS ((‘Performance‐Enhancing Substance*’ OR ‘Performance‐Enhancing drug*’ OR ‘cognitive doping’ OR ‘image and performance‐enhancing’ OR doping) AND (sport* OR athlete*)) AND (LIMIT‐TO (LANGUAGE, ‘English’))”. On Scopus, the key terms were optimized to maximize the number of retrieved documents while reducing the noise from non‐relevant publications. We limited the search to documents in English in order to retrieve only publications from the standard international scientific literature.^24^ A total of 4330 relevant documents were collected for the analysis. The collected literature spanned between 1948 and 2023. The sample was initially analyzed using the *bibliometrix* package for R^25^ to provide insight into the structure of knowledge in the literature on performance enhancers in sports.

### 2.2 Data eligibility

The scientometric analysis was conducted by importing the documents and their references into Citespace (version 6.1.R6, 64‐bit).^26^ Altogether, the documents retrieved from Scopus cited 200,201 references. Of these, a total of 193,076 references (96.44%) were labeled as valid for further analysis.

In scientometric studies, a data loss of 1.00–5.00% is common, and it is caused by inconsistencies in the citation format.^17^, ^27^ The data loss of the current work (i.e., 3.56% of the total references) is, therefore, acceptable and in line with the standards of scientometric reviews. For the analysis, CiteSpace's “Remove Alias” function was turned on to eliminate repeated or identical entries during the analysis.

### 2.3 Document co‐citation analysis (DCA)

**FIGURE 1:**
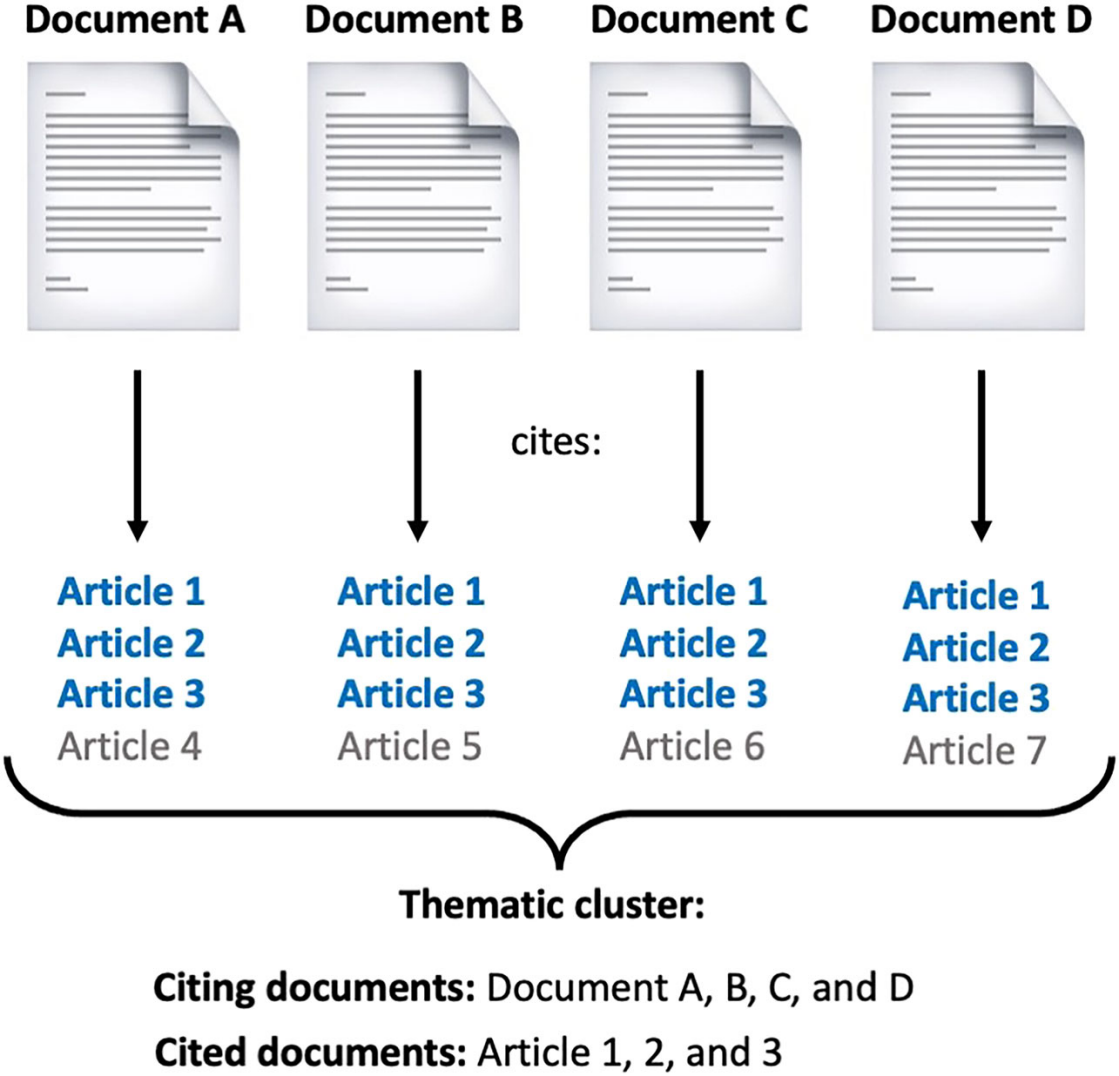
Identification of the thematic clusters of research through the document co‐citation analysis (DCA).

To identify the major domains of research in the literature on performance enhancers in sports, we conducted a DCA. DCA relies on the frequency of co‐citation (i.e., instances in which two or more documents are cited together by other publications) between documents.^26^, ^28^, ^29^ The general assumption behind DCA is that documents with a shared thematic interest will be frequently cited together by other documents. By modeling the single documents as nodes, the co‐citations as links, and the frequency of co‐citations as link weights, DCA generates a network of connected documents. The obtained DCA network includes both citing (i.e., documents retrieved directly from Scopus) and cited documents (i.e., documents cited by the publications collected from Scopus). Figure 1 depicts the principles behind the DCA and how clusters are identified.

**FIGURE 2:**
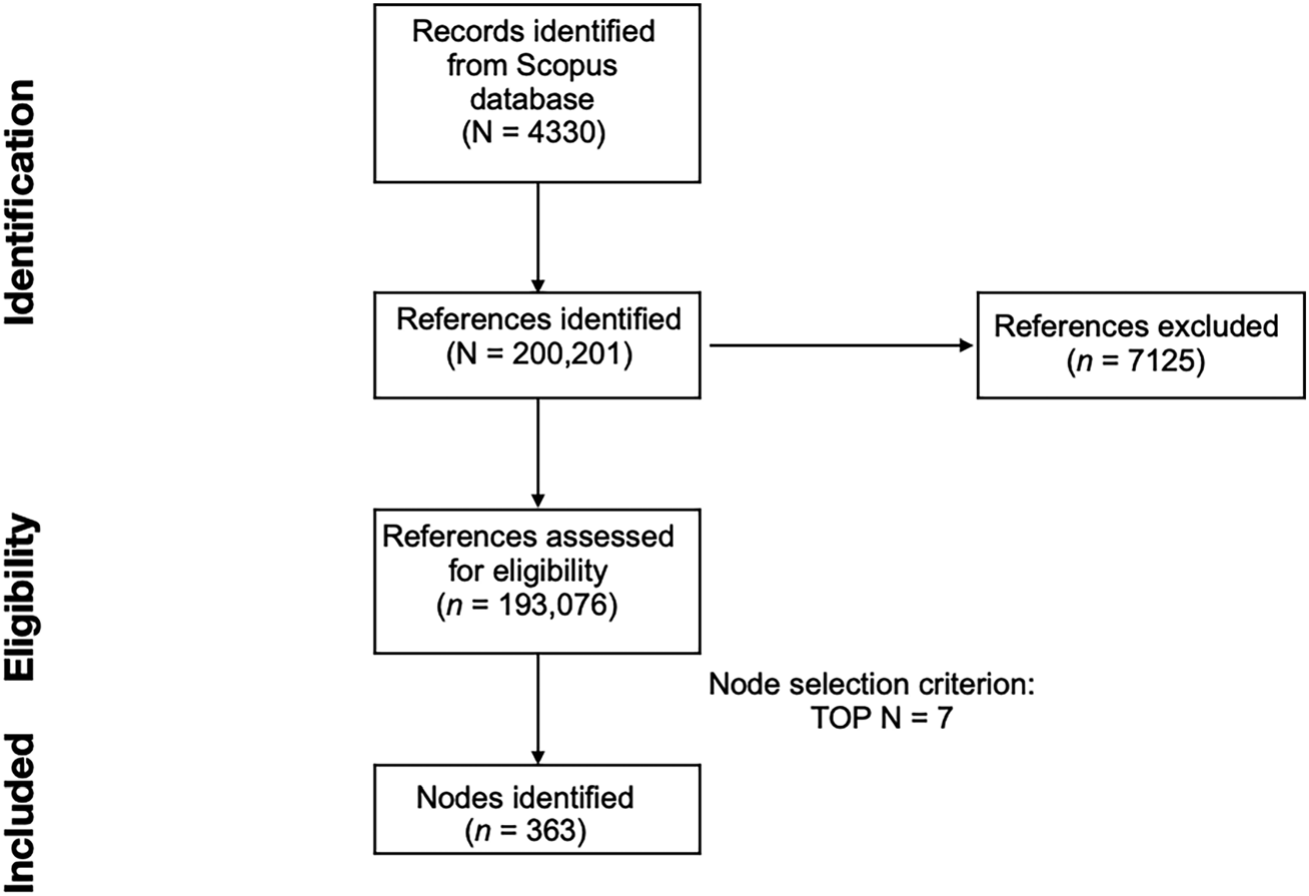
Preferred reporting items for systematic reviews and meta‐analyses (PRISMA) flowchart for literature search, reference eligibility, and inclusion.

Several node selection criteria are available on CiteSpace to optimize the DCA network. Among the available node selection criteria, the *g*‐index, TOP *N*, and TOP *N*% are the most common. The *g*‐index is derived from the *h*‐index, and it is the “largest number that equals the average number of citations of the most highly cited *g* publications.”^30^, ^31^, ^32^, ^33^ TOP *N* includes in the network the *N* most cited documents by each time slice (i.e., 1 year for the current paper). TOP *N*% includes in the network the N% most cited references for each time slice. Each node selection criterion is combined with a scaling factor value. The scaling value sets the threshold for the node selection criterion and allows the user to control the number of documents included in the final network. Following the approach of our previous publications (e.g., Yee et al.^17^), we compared the DCA networks generated using *g*‐index with *k* set to 15, 25, 50, and 75; TOP *N* with *N* set to 5, 7, 10, and 50; and TOP *N*% with *N*% set to 1 and 10. All networks were compared in terms of their structural properties, and only the one with the most optimal structure was selected for the analysis. The DCA generated with TOP *N* with scaling factor *N* set to 7 was chosen. Figure 2 reports the methodological steps of the study, from the literature search to the reference inclusion.

### 2.4 Metrics

The results of this study will be presented using both structural and temporal metrics.

Modularity, silhouette, and betweenness centrality are available structural metrics on CiteSpace. Modularity is a network‐level metric, with values ranging from 0 to 1. Modularity provides a measure of the extent to which the network is divisible into separate clusters.^34^ Higher values of modularity are an index of higher divisibility into distinct clusters.^35^

Conversely, the silhouette score is a cluster‐level metric that ranges from −1 to 1. Higher silhouette values are obtained by clusters with high internal consistency and separation from other clusters.^36^ Betweenness centrality is a node‐level metric that measures the influence of a single node on the overall network. Values of betweenness centrality range from 0 to 1, with higher values obtained by nodes that often connect other random pairs of nodes.^27^, ^37^

Temporal metrics include citation burstness and sigma. Citation burstness is a node‐level metric that indicates an abrupt increase in the number of citations received by an article. Values for the citation burstness are computed through Kleinberg's algorithm.^38^ Citation burstness allows detecting the impactful documents that have received significant attention from experts in the field.^39^ Sigma takes into account both the betweenness centrality and the citation burstness of a node, and it is computed with the equation (centrality + 1)^*burstness*^. By combining both structural and temporal metrics, sigma is and indicates the novelty of a document and its influence on the overall network.^35^, ^40^

## 3 RESULTS

### 3.1 Bibliometric analysis on the citing documents

The sample of documents grew from 1948 to 2023 with an annual growth rate of 8.03%. Each document obtained an average of 19.57 citations with an average document citation by year of 1.71. The documents cited more often were authored by Bassett and Howley^41^ (total citations = 1330; total citations per year = 55.42), by Graham^42^ (total citations = 573; total citations per year = 24.91), and by Simonsen et al.^43^ (total citations = 380; total citations per year = 31.67).

**FIGURE 3:**
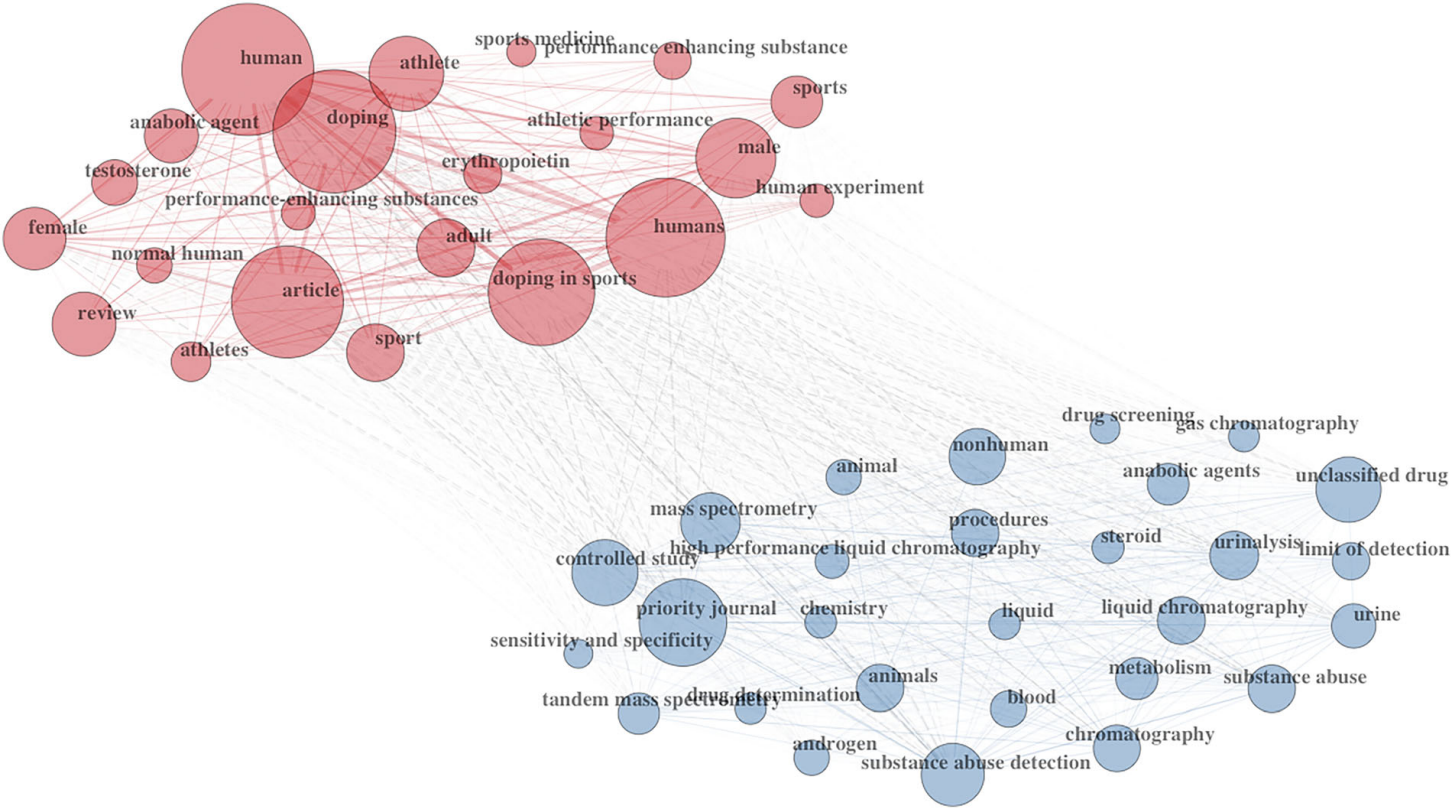
Top 50 keywords co‐occurrences.

An amount of 6244 keywords selected by the authors indexed the documents. The most popular keywords plus were *doping* (*n* = 1038 documents), *sport* (*n* = 351 documents), *doping control* (*n* = 199 documents), *anti‐doping* (*n* = 192 documents), *mass spectrometry* (*n* = 172 documents), *sports* (*n* = 142 documents), *athletes* (*n* = 123 documents), *erythropoietin* (*n* = 100 documents), *urine* (*n* = 100 documents), and *anabolic steroids* (*n* = 94 documents; see Figure 3).

**FIGURE 4:**
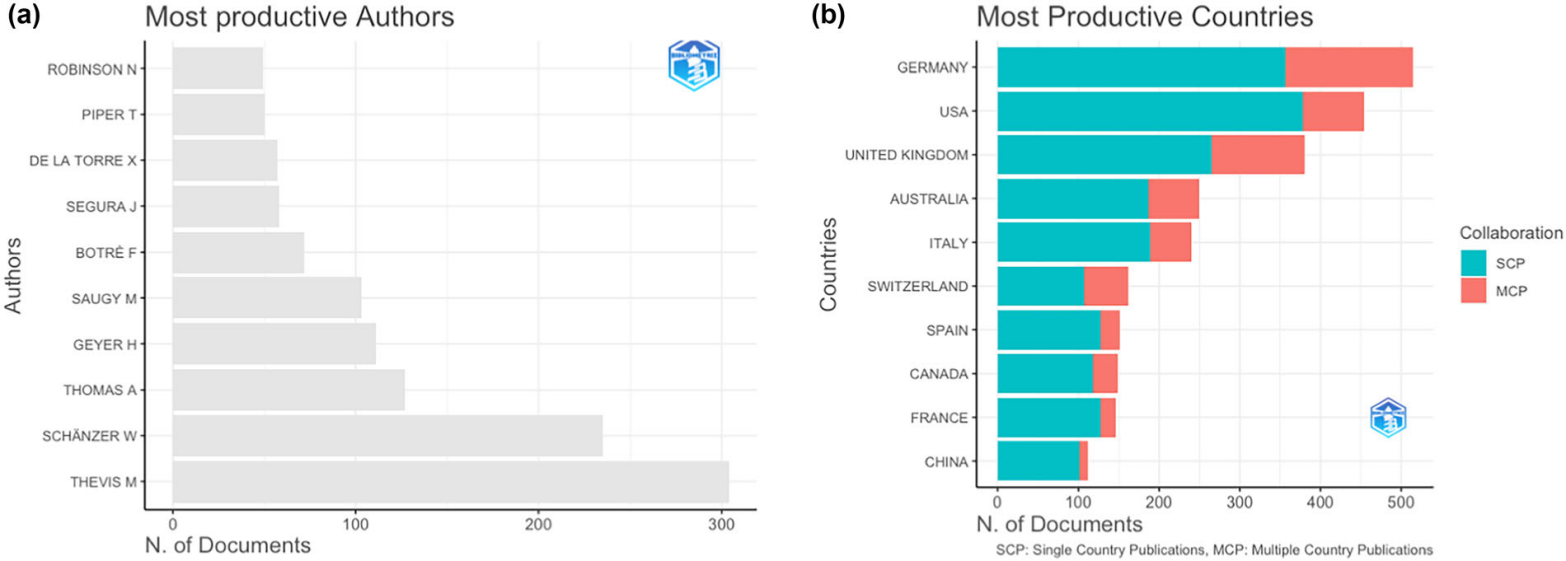
Results of the bibliometric analyses. Images are generated using *bibliometrix* package for R.^25^ (a) Ten most productive countries. (b) Ten most occurring countries in authors' affiliations.

The documents were authored by a total of 8950 unique authors. On average, the dataset included 0.484 documents per author and an average of 3.96 co‐authors per document. The two most productive authors in the sample were M. Thevis (*n* = 304 documents) and W. Schänzer (*n* = 235 documents; see Figure 4a).

Authors' affiliations were mostly from Germany (*n* = 514 documents; frequency = 0.1411; Single Country Publications [SCPs] = 357; Multiple Country Publications [MCPs] = 157), the United States of America (*n* = 454 documents; frequency = 0.1247; SCP = 378; MCP = 76), or from the United Kingdom (*n* = 380 documents; frequency = 0.1043; SCP = 265; MCP = 115; see Figure 4b).

The main sources in the literature on doping were *Drug Testing and Analysis* (*n* = 399 documents), the *British Journal of Sports Medicine* (*n* = 79 documents), and *Performance Enhancement & Health* (*n* = 76 documents).

### 3.2 Properties of the DCA network

**FIGURE 5:**
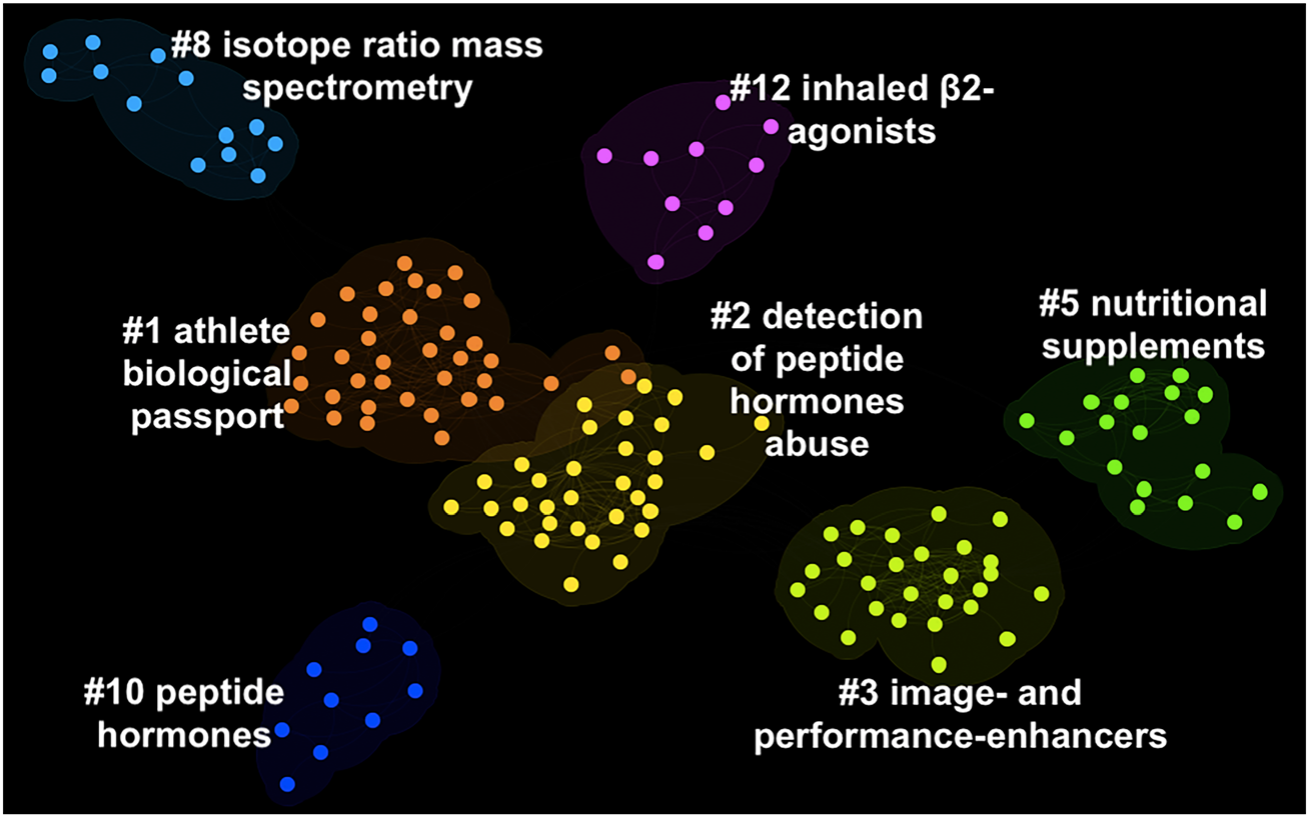
Document co‐citation analysis network of the literature on doping. The network represents the existing literature on doping by using documents as nodes and co‐citation patterns as edges. The seven major clusters are grouped by color. The image was generated with CiteSpace software.^26^

The optimal DCA generated a network with 363 nodes (i.e., documents) and 1092 links. Therefore, each document was, on average, connected to another 3.01. The network had a modularity of 0.8209 and an average silhouette score of 0.9489. Thus, the network was highly divisible into consistent and separate clusters (i.e., thematic domains of research). The network is represented in Figure 5.

### 3.3 Major thematic clusters of research

Seven major thematic clusters of research were detected in the DCA network. All clusters were automatically labeled using CiteSpace's log‐likelihood ratio (LLR) algorithm. As compared with other automated methods (e.g., mutual information and latent semantic information), LLR tends to provide the best results in terms of uniqueness and coverage of cluster‐related topics.^27^, ^44^ However, we also conducted a qualitative inspection of the clusters' content to assess the accuracy of the LLR label. As in our previous publications (e.g., Yee et al.^17^), when the LLR label was not representative of a cluster's content, we labeled the cluster manually.

In the network, the three largest clusters were cluster #1 (size = 37 documents; silhouette = 0.951; mean publication year = 2005), cluster #2 (size = 32 documents; silhouette = 0.921; mean publication year = 2001), and cluster #3 (size = 27 documents; silhouette = 0.91; mean publication year = 1998). The three clusters with the highest silhouette scores were cluster #10 (size = 10 documents; silhouette = 0.996; mean publication year = 1995), cluster #8 (size = 13 documents; silhouette = 0.986; mean publication year = 2010), and cluster #12 (size = 10 documents; silhouette = 0.982; mean publication year = 2004). Metrics for all the seven major thematic clusters of research are presented in Table 1.

**TABLE 1:** Metrics of the seven thematic clusters of research. For each cluster, the number of included documents (i.e., size), the silhouette score, the mean publication year, the log‐likelihood ratio (LLR) label, and the suggested label are reported.

ID	Size	Silhouette	Mean year	LLR label	Suggested label
1	37	0.951	2005	Recombinant human erythropoietin	Athlete biological passport
2	32	0.921	2001	Recombinant human erythropoietin	Detection of peptide hormones abuse
3	27	0.910	1998	Performance‐enhancing substance	Image and performance enhancers
5	18	0.981	1997	Nutritional supplements	Nutritional supplements
8	13	0.986	2010	Endogenous urinary steroids	Isotope ratio mass spectrometry
10	10	0.986	1995	GH withdrawal	Peptide hormones
12	10	0.982	2004	Critical appraisal	Inhaled *β*2‐agonists

### 3.4 Most impactful documents

In the network, a total of 51 documents obtained a significant burst in their citation history. However, of the 51 documents, six were repeated entries that were not recognized by the software. In case of a repeated entry, we only considered the document with the highest value for the citation burstness among the two. The three documents with the highest citation burstness were authored by Ntoumanis et al.^45^ (citation burstness = 25.66; duration = 6 years), by Lasne and Ceaurriz^46^ (citation burstness = 17.66; duration = 7 years), and by Catlin Don et al.^47^ (citation burstness = 17.61; duration = 4 years). The metrics for the 10 documents with the strongest citation burstness are reported in Table 2.

**TABLE 2:** Metrics for the 10 documents with the highest value of citation burstness.

Reference	Citation burstness	Publication year	Burst begin	Burst end	Duration (years)	Sigma	Centrality
Ntoumanis Nikos, Ng Johan YY, Barkoukis Vassilis, Backhouse Susan. Personal and psychosocial predictors of doping use in physical activity settings: a meta‐analysis *Sports medicine*. 2014;44:1603–1624^45^	25.66	2014	2017	2023	6	1.78	0.02
Lasne Françoise, Ceaurriz Jacques. Recombinant erythropoietin in urine *Nature*. 2000;405:635–635^46^	17.66	2000	2001	2008	7	6.74	0.11
Catlin Don H, Sekera Michael H, Ahrens Brian D, Starcevic Borislav, Chang Yu‐Chen, Hatton Caroline K. Tetrahydrogestrinone: discovery, synthesis, and detection in urine *Rapid Communications in Mass Spectrometry*. 2004;18:1245–1,049.^47^	16.61	2004	2008	2012	4	1.88	0.04
Lentillon‐Kaestner Vanessa, Carstairs Catherine. Doping use among young elite cyclists: a qualitative psychosociological approach *Scandinavian Journal of Medicine & Science in Sports*. 2010;20:336–345.^48^	16.20	2010	2013	2017	4	1.22	0.01
Lazuras Lambros, Barkoukis Vassileios, Rodafinos Angelos, Tzorbatzoudis Haralambos. Predictors of doping intentions in elite‐level athletes: a social cognition approach *Journal of Sport and Exercise Psychology*. 2010;32:694–710^49^	14.15	2010	2012	2018	6	1.71	0.04
De Hon Olivier, Kuipers Harm, Van Bottenburg Maarten. Prevalence of doping use in elite sports: a review of numbers and methods *Sports medicine*. 2015;45:57–69.^50^	13.88	2015	2017	2023	6	1.36	0.02
Lasne Françoise, Martin Laurent, Crepin Nathalie, Ceaurriz Jacques. Detection of iso‐electric profiles of erythropoietin in urine: differentiation of natural and administered recombinant hormones *Analytical biochemistry*. 2002;311:119–126.^51^	13.82	2002	2004	2009	5	2.59	0.07
Sharpe Ken, Ashenden Michael J, Schumacher Yorck O. A third generation approach to detect erythropoietin abuse in athletes *Haematologica*. 2006;91:356–363.^52^	12.12	2006	2009	2012	3	2.24	0.07
Bloodworth Andrew, McNamee Michael. Clean Olympians? Doping and anti‐doping: The views of talented young British athletes *International journal of drug policy*. 2010;21:276–282.^53^	9.84	2010	2013	2017	4	1.04	0.00
World Anti‐Doping Agency. WORLD ANTI‐DOPING CODE INTERNATIONAL STANDARD PROHIBITED LIST 2021https://www.wada‐ama.org/sites/default/ files/resources/files/2021list_en.pdf 2021.^54^	9.26	2021	2021	2023	2	1.00	0.00

## 4 DISCUSSION

The current manuscript has the goal to reflect upon the hot topics of interest in the community with a particular focus on anti‐doping in sport. To do so, we conducted a scientometric review of the whole literature on performance‐enhancing drugs in sports. With this approach, we aimed to *(i)* analyze the structure of knowledge (most active countries, journals, authors, and most frequently used keywords); *(ii)* identify the most impactful documents; and *(iii)* pinpoint the main domains of research in the literature on performance enhancers in sports.

From the analysis, it emerged that the scientific knowledge on performance‐enhancer drugs in sports was strongly fostered by German institutions and by the journal *Drug Testing and Analysis*. *Drug Testing and Analysis* aims to publish articles that focus on the detection of illicit and emerging substances. The journal's scope aligns with the large effort that has been directed to improve detection methods for performance‐enhancing drug use. The most prolific author in the field emerged to be Mario Thevis, and the most occurring keywords relate to anti‐doping approaches and the effort towards the development of detection methods (e.g., mass spectrometry) as pivotal fields in the literature.

Overall, the literature on performance‐enhancing drugs in sport was strongly influenced by the publication of the works by Ntoumanis et al.,^45^ Lasne and Ceaurriz,^46^ and Catlin Don et al.,^47^ as well as the documents by Bassett and Howley,^41^ Graham,^42^ and Simonsen et al.^43^

On the basis of the patterns of co‐citation among published documents, we identified seven main domains of research in the literature. Each of the seven domains of research is reviewed in the following subsections. The thematic clusters of research are discussed chronologically, following the average year of the documents' publication, from the earliest to the most recent. For each cluster, the main citing documents will be discussed together with their coverage (i.e., the number of cited documents in the cluster) and Global Citation Score (GCS; i.e., the number of citations received on Scopus).

### 4.1 Cluster #10: Peptide hormones

The earliest cluster in the network was cluster #10. Here, the first three major citing documents were authored by Wallace et al.^55^ (coverage = 4; GCS = 176), Birkeland and Hemmersbach^56^ (coverage = 4; GCS = 26), and by Jenkins^57^ (coverage = 3; GCS = 14). This cluster of research regards the use of peptide hormones, such as the growth hormone and erythropoietin, among athletes to enhance their performances. In the late 1900s, the use of peptide hormones as performance enhancers became popular among athletes. The reason behind this trend lies in the increased efficacy of detection methods for anabolic agents (e.g., steroids), which, in turn, pushed some competitors to find alternative ergogenic aids. Documents in the cluster studied the enhancing effects of peptide hormones and their negative health consequences.^55^, ^57^ Furthermore, documents pointed out the difficulties in detecting the abuse of peptide hormones from urine samples and suggested that blood analyses might represent a promising detection matrix.^56^ Interestingly, in the cluster, the most cited work was by Leif et al.^58^ In this study, the authors evaluated a novel method to detect administered recombinant human erythropoietin from blood and urine specimens. This method was based on the observation that recombinant human erythropoietin has a less negative electric charge as compared with endogenous erythropoietin.

### 4.2 Cluster #5: Nutritional supplements

The second earliest group of documents in the network is cluster #5. The three major citing documents of the cluster were authored by Pipe and Ayotte^59^ (coverage = 9; GCS = 107), Bouchard et al.^60^ (coverage = 7; GCS = 38), and Kohler and Lambert^61^ (coverage = 5; GCS = 31).

This cluster contains documents pertaining to the intersection between doping and dietary supplement use among athletes. It is noteworthy that many of these supplements contain substances banned in sports, associated with significant health risks, as highlighted by Pipe and Ayotte.^59^ In alignment with this objective, Bouchard et al.^60^ devised a seven‐stage model to facilitate informed decision making for individuals employing or contemplating the use of stimulants in sports.

Moreover, the problematic use of dietary supplements extends beyond the realm of sports. For instance, Haller and Benowitz^62^ reported the adverse cardiovascular and central nervous system events associated with dietary supplements containing ephedra alkaloids. At the time of the publication, these substances were popular in the United States as a means of losing weight and increasing energy. Similarly, Bell and Jacobs,^63^ Nehlig and Debry,^64^ and Tarnopolsky^65^ examined the effect of caffeine on physical performances.

### 4.3 Cluster #3: Image and performance enhancers

The third cluster in chronological order was cluster #3. In the cluster, the major citing documents were authored by Tokish et al.^66^ (coverage = 11; GCS = 134), Ambrose^67^ (coverage = 10; GCS = 46), and by Boyce^68^ (coverage = 7; GCS = 9). The documents in the cluster presented the most common performance‐enhancing substances used by athletes. The documents encompassed a variety of more or less available substances, such as anabolic steroids, growth hormone, erythropoietin, caffeine, and creatine (e.g., Thiblin et al.^69^). Connecting with the thematic interest of the previous cluster, some cited documents investigated the hormonal and physiological effects of androstenedione, the major precursor of testosterone that is available without prescription (e.g., previous studies^70^, ^71^, ^72^, ^73^). Among the most popular performance enhancers, the most used ones at the end of 1900s were the anabolic steroids.^74^ Dickinson et al.^75^ and Melia et al.^76^ have observed a rising trend in hormone abuse among adolescents and adults. This upward trajectory is particularly pronounced within the high school female student population, as noted by Dickinson et al.^75^ The increasing prevalence of hormone consumption among female adolescents can be attributed to the fact that several hormonal substances are employed by non‐athletes to enhance their body image.^77^ Dickinson et al.^75^ argued that anabolic steroid use among females might be considered as a subset of disordered eating habits and physique‐altering drug abuse.

### 4.4 Cluster #2: Detection of peptide hormones abuse

The following thematic domain was cluster #2. In the cluster, the major citing documents were authored by Thevis and Schanzer^78^ (coverage = 10; GCS = 24), Rigamonti et al.^79^ (coverage = 8; GCS = 27), and by Dickinson et al.^75^ (coverage = 7; GCS = 14). The central topic of the cluster regards the issue of detecting the abuse of peptide hormones in sports (e.g., Lasne and Ceaurriz^46^). In fact, the large use of the growth hormone in sports could be attributed to several factors, including the lack of a reliable method for detecting it in body fluids. For this reason, the development of reliable detection methods was of paramount importance. To do so, researchers developed various strategies for the detection of these substances in blood or urine specimens using different biochemical techniques (e.g., immunoaffinity purification, isoelectric focusing, and gel electrophoresis). Specifically, Rigamonti et al.^79^ reviewed the available and novel methods to assess plasma levels of growth hormone biomarkers. Growth hormone‐sensitive substances are more stable than the hormone itself and allow for more stable and reliable assessments.

### 4.5 Cluster #12: Inhaled β2‐agonists

The following thematic cluster of research is cluster #12. The major citing documents were authored by Kayser et al.^80^ (coverage = 4; GCS = 168), Furlanello et al.^81^ (coverage = 3; GCS = 57), and by Matich^82^ (coverage = 3, GCS = 9). Many documents in the cluster focused on studying the enhancing effects of inhaled *β*2‐agonists, which are frequently used in anti‐asthmatic therapy. Because *β*2‐agonists have ergogenic effects, they were banned by the International Olympic Committee. However, the enhancing effect of inhaled *β*2‐agonists was not clear and debated across studies.^83^, ^84^

### 4.6 Cluster #1: Athlete biological passport

The major citing documents in cluster #1 were authored by Bowers^85^ (coverage = 8; GCS = 21), Thevis^86^ (coverage = 7; GCS = 59), and by Sottas et al.^87^ (coverage = 6; GCS = 173). The documents in the cluster discussed the analytical advancements in the field of doping detection. Specifically, Bowers^85^ provides an overview of the techniques that allowed for the enhancement of detection methods. For instance, the use of liquid chromatography/tandem mass spectrometry and gas chromatography/combustion/isotope‐ratio mass spectrometry has been crucial for developing methods for detecting of anabolic agents. Similarly, methods for detecting proteins and peptides have benefited from liquid chromatography/tandem mass spectrometry. With these analytical advancements in the background, a new paradigm for drug testing emerged: the Athlete Biological Passport. The Athlete Biological Passport is based on the personalized monitoring of biomarkers of doping, with the advantage of being independent of the ever‐increasing and ever‐changing drug market.^87^, ^88^ The assumption behind the Athlete Biological Passport is that doping substances trigger physiological changes, which are responsible for the enhancing effect. Therefore, specific biomarkers of such physiological changes can help the detection of doping abuse. For this reason, many documents in the clusters started to direct their research effort in identifying specific biomarkers of doping use (e.g., Pottgiesser et al. and Teale et al.^89^, ^90^).

### 4.7 Cluster #8: Isotope ratio mass spectrometry

The most recent cluster in the network was cluster #8. Here, the major citing documents were authored by Piper et al.^91^ (coverage = 4; GCS = 29), Thevis et al.^92^ (coverage = 4; GCS = 12), and Schumacher et al.^93^ (coverage = 3; GCS = 67). The documents in the cluster discussed the potential strategies to improve the accuracy of doping detection using isotope ratio mass spectrometry. For instance, Cawley and George^94^ and Piper et al.^91^, ^95^ showed how carbon isotope ratios in urine specimens can help detect exogenous anabolic steroid abuse. This cluster of research represents the natural prosecution of the work presented in cluster #2 about the methods for detecting peptide hormone abuse.

### 4.8 Limitations of the study

The present study employs a scientometric approach to identify impactful documents and prevalent trends in the scientific literature on doping. Although this method has proven valuable for achieving our research objectives, it is essential to acknowledge certain limitations. Firstly, the outcomes of the scientometric analysis are heavily contingent on the data utilized as input. In our case, we collected all available literature on doping from Scopus. Therefore, some relevant publications that are not indexed in Scopus (e.g., older publications) and that have not been cited by the documents retrieved from Scopus might have not been collected. Moreover, despite our efforts in employing a diverse set of key terms commonly associated with doping substances, there is a possibility that some relevant terms were inadvertently omitted. This limitation is particularly noteworthy given the ever‐expanding group of substances utilized to enhance individuals' image or physical performance. Notably, a considerable proportion of these performance‐enhancing substances remains unidentified by doping authorities and experts in the field. Secondly, the present study employs a quantitative approach to analyze co‐citation patterns among documents.

Given its reliance on citation patterns, the scientometric analysis provides a more comprehensive understanding of past research trends and publications, in contrast to the ongoing emergence of more recent ones in the literature (e.g., NPS as doping).

## 5 CONCLUSION

The current manuscript outlined the structure of knowledge around doping in sport. In doing so, the most impactful documents as well as the main research thematic domains were identified and discussed. With the general aim of informing anti‐doping authorities and helping the design of proper anti‐doing policies, the literature strongly focused on identifying the substances that might confer an unfair performance enhancement as well as the ones that might represent serious hazards for the athletes. Alongside the identification of new substances, the scientific community has made enormous progress in the methods for detecting doping abuse. However, although technical and analytical methods for doping detection are advancing at a rapid pace, new ways to dope are continuously introduced in sports. It is the example of genetic doping^96^ and NPS used as performance enhancers.^18^ It is for this reason that the anti‐doping community has strengthened its educational campaigns to inform sports figures and make them responsible for the health risks posed by doping and the importance of doping control processes.^96^

## AUTHOR CONTRIBUTIONS

Conceptualization: AC, OC, OR, GE; methodology: AC; formal analysis: AC; investigation: AC; writing—original draft preparation: AC, OC, MM, NS, OR, GE; writing—review and editing: AC, OC, MM, NS, OR, GE; supervision: GE. All authors have read and agreed to the published version of the manuscript.

## CONFLICT OF INTEREST STATEMENT

The authors declare no conflict of interest.

## Data Availability

Data will be available upon request to the corresponding author.
